# Core values and best practice criteria for interprofessional teams in primary care: a qualitative interview study with general practitioners and other health professionals from Bavaria, Germany

**DOI:** 10.1186/s12875-025-03114-3

**Published:** 2025-12-11

**Authors:** Katharina Zeiser, Dominik Weissenburger, Michaela Trompke, Lisa Schumacher, Simon Schwill, Wolfgang Ritter, Marco Roos

**Affiliations:** 1 General Practice, Institute of General Practice and Health Services Research, University Hospital of Augsburg, Augsburg, Germany; 2 Department of General Practice and Health Services Research, University Hospital Heidelberg, Heidelberg, Germany; 3 Bavarian Association of General Practitioners, Munich, Germany

**Keywords:** General practice, Primary care, Interprofessional collaboration, Team-based care, Qualitative research

## Abstract

**Background:**

The German healthcare system is confronted with a shortage of general practitioners (GPs) due to demographic changes and an aging workforce. Concepts such as team-based care, which ensure high-quality primary care, are necessary to address these future challenges. This study aimed to identify values as well as best practices of such team-based concepts.

**Methods:**

We conducted *n* = 15 individual interviews with health professionals primarily working in primary care settings, including GP trainees, employed or self-employed GPs, medical assistants, primary care management or physician assistant students, and other health professionals (mean age = 36.13 years, 66.67% female). The interviews were transcribed verbatim and coded using a deductive category system based on prior research. For data analysis, we used qualitative content analysis following the framework method.

**Results:**

Participants emphasized patient-centred and continuous care as core values of primary care, highlighting the importance of establishing trusting relationships through sufficient time with patients. In this context, they rated interprofessional team-based care as particularly beneficial for patients who are chronically ill and disadvantaged. The participants supported primary care models characterized by GP-centredness and gatekeeping, a high degree of digitalization, cooperation with non-physician health professionals, and well-defined roles within interprofessional teams. They also stressed the importance of remuneration and work-life balance. To evaluate future concepts of primary care, the interviewees recommended using both staff- and patient-reported measures, as well as operational metrics.

**Conclusions:**

Our results indicate that the core values of primary care, such as patient-centredness and continuity of care, may be enhanced through interprofessional teamwork. While these values contribute to the intrinsic motivation of high-quality care, structural factors such as fair remuneration and digitalization are crucial for effective practice. To evaluate care models, the patient perspective, along with staff satisfaction and team performance, is regarded as an essential outcome measure.

**Supplementary Information:**

The online version contains supplementary material available at 10.1186/s12875-025-03114-3.

## Background

Primary care should be built upon core values that serve as guiding principles for ethical and clinical decision making across all professions involved in the primary care setting [1]. The European definition of general practice/family medicine emphasizes the provision of primary, continuous, and person-centered care, which incorporates the physical, psychological, social, and cultural aspects of health [2]. This vision defines the profession’s priorities and shapes the quality and integrity of patient care. Based on a qualitative study, Arvidsson, et al. [1] identified that the core values of general practice (GP) in Europe primarily emphasise patient-centred care, continuity of care, community orientation, interdisciplinary collaboration, and evidence-based practice [1].

Reflecting on values in primary care is essential not only for promoting cost-effective and high-quality care but also for ensuring that medical practices align with ethical principles and address the needs of patients. These values often interrelate, creating a comprehensive framework. For example, evidence-based medicine should not be understood merely as a technical or rule-based approach, but must also incorporate patient-centred perspectives to ensure patients’ care needs [3].

The delivery of value-based primary care in Germany and other European countries is threatened by an impending shortage of general practitioners (GPs), driven by demographic changes and an aging medical workforce [4]. At the same time, an aging society leads to a greater need for managing multimorbidity [5]. Those changes could lead to an inadequate healthcare provision, especially in rural areas [4, 6, 7]. A specific challenge in the German county of Bavaria is the coexistence of large urban areas with mostly high physician density and very rural regions with low density [8], but even in some urban areas, a shortage of GPs is anticipated [8]. These aspects have to be addressed in terms of planning healthcare for an aging population and the impending shortage of GPs. Moreover, the lack of interprofessional approaches in outpatient care, combined with the strict separation of outpatient and inpatient care in Germany, hampers coordination and creates gaps in the care process [9]. Therefore, there is a need for new concepts of care to ensure the delivery of high-quality, cost-effective, and value-based primary care.

Among the strategies to address future challenges, interprofessional teams and the strengthening of task shifting are considered promising approaches [7, 10]. In Germany, GP/family medicine predominantly involves GPs and medical assistants (MA). In some practices, there are also specialized MAs (so-called VERAH/NäPa) or assistants with academic qualifications, such as primary care managers or physician assistants. Other team members are rarely associated with GP. The promotion of stronger interprofessional teamwork and greater task-shifting within the primary care setting is currently a key focus of discussion [11]. In countries such as the United States, Australia, the United Kingdom, Sweden, Canada, and the Netherlands, patients have reported highly positive experiences with interprofessional teams in primary care, and several studies have also indicated promising results regarding cost-related outcomes [12, 13]. In Germany, pilot studies and interventions have also shown promising results for team-based care models in reducing hospitalisation rates and costs among patients with chronic illnesses [14].

However, aligning innovative care models with the core values of primary care is essential, as both the implementation of new models and a values-based approach to care are considered key strategies for addressing the complex challenges associated with an aging population [10, 15]. Interprofessional collaboration holds particular potential to strengthen comprehensive, patient-centred care, especially in the management of chronic conditions [16]. At the same time, the shift towards interprofessional team-based care often involves digitalisation, increased workloads, and changes in professional roles—factors that may risk fragmenting care and weakening continuity [1]. Therefore, the potential impact of new care models on care quality and values-based delivery must be carefully evaluated to avoid unintended consequences, such as reduced continuity of care.

Best practice criteria for interprofessional team-based care in Germany could facilitate the implementation of this new concept in a way that prevents unintended negative consequences. A ‘best practice’ refers to an effective, evidence-based intervention applied in a particular real-world setting [17]. Based on this definition, we understand best practice criteria as standards or examples used to identify and evaluate effective and value-based concepts, processes, or interventions regarding interprofessional team-based primary care.

Our study aimed to explore the core values of primary care from the perspective of GPs and other health professionals in Bavaria, Germany, as well as to identify best practice criteria for interprofessional team-based care. In addition, we sought to determine how these best practice criteria can be measured for evaluating care concepts, particularly in terms of alignment with primary care core values.

## Methods

To explore values and best practice criteria, we conducted individual interviews that were embedded in a mixed-methods approach with additional focus groups and a quantitative survey [18]. For the interviews, we selected professional groups that are involved in primary care. Guided by purposive sampling principles – such as ensuring representation from each professional group – we preferentially selected participants who demonstrated strong engagement with and interest in team-based concepts, thereby maximizing the depth and relevance of the data collected. Persons who had shown a high level of preparedness to contribute to innovative ideas in the focus groups that took place beforehand were therefore preferred to be part of the individual interviews. In the same manner, persons with experience in other concepts of care or with a high interest were picked preferentially. We contacted at least one person from each group that participated in the focus group study. Of the 21 individuals contacted, 15 agreed to participate, while five did not respond within the given timeframe and one declined participation.

To strengthen trustworthiness of the qualitative data, we implemented several measures. The credibility of the findings was supported through peer debriefing during the pretesting of the interview guideline. To foster the interpretability and transferability of findings, the interviews began with questions about the participants’ professional experience and social life. The main part of our guideline for the individual interviews contained questions about good general practice/family medicine (e.g. *‘What do you understand by good general practice/family medicine?’*,* ‘What qualities or features should a future general practice have*,* in your opinion?’*) as well as questions about experience with concepts of primary care in Germany and abroad, that could possibly be regarded as best practice (e.g. *‘Have you encountered other concepts of primary care abroad or in other regions of Germany? If yes*,* which ones? Could you briefly describe them?’*) (see Additional file 1).

The interviews were conducted between July 2, 2024, and July 26, 2024, using the video conferencing tool *Zoom*. Each interview was conducted by two of the four research team members, who alternated between moderating the interviews and taking minutes. The audio recordings were captured using an offline device, then transcribed verbatim using *NoScribe* software, and double-checked by a research team member against the audio. Another team member verified that the transcripts were correctly pseudonymized and in accordance with the transcription guidelines of Kuckartz and Rädiker [19]. The transcriptions were in standard German, without dialects or accents. Dependability and confirmability were promoted by systematic documentation and the involvement of two researchers of the team in both the verification of transcription accuracy and pseudonymization and the following coding process.

The qualitative content analysis of the data followed the framework method [20] with *MAXQDA* software (version 2022). Every single coding of the interviews was analysed in each case by a physician and a health scientist in order to consider both perspectives. Researcher reflexivity was further fostered through regular team discussions within this multidisciplinary team, ensuring that assumptions and potential biases were continuously reflected throughout data collection and analysis.

Analysis followed a deductive category system based on prior research (see Additional file 2 for relevant segments of the coding tree). Codes were sorted in *Excel* (version 2408) for category- and case-specific summaries.

For the presentation of results regarding values in general practice, we categorized the answers into core values, methods, and competencies necessary for implementing those values, as well as the expected outcomes reported by the study participants.

All participants provided written informed consent before their inclusion in the study. The project (number 24–0057), in which the interviews were embedded, was submitted to the Medical Faculty of Ludwig Maximilian University of Munich on January 19, 2024, and received ethics approval on March 18, 2024.

Further methodological details are provided in the COREQ checklist (see Additional file 3).

## Results

### Description of the sample characteristics

A total of 15 persons participated in the individual interviews. Each interview session lasted between 20 and 60 min. At least one person participated from each of the professional groups, including GP trainees, employed GPs, self-employed GPs, PA students, PCM students, MPAs, and other healthcare professionals. A total of *n* = 10 participants were female, and *n* = 5 participants were male. The age ranged from 27 to 45 years (mean 36.13 years, SD 6.13). The respondents had six months to over 20 years of professional experience in various medical or medical-related fields. They worked predominantly in GP practices, although the size varied. Both rural and urban practices were included. A few interviewees worked in clinics or aid organizations. They were employed either full-time or part-time, and their tasks included patient contact in face-to-face or online consultations, home visits, team meetings, and administrative work. Most interviewees were in a partnership and had children. An overview of the socio-demographic characteristics of the participants is shown in Table 1.

**Table 1:** Sociodemographic characteristics of the interviewees

Interview number	Interview duration (minutes)	Group	Gender	Age
1	40	GP trainee	Female	35
2	50	Employed GP	Male	38
3	35	GP trainee	Male	39
4	35	GP trainee	Female	27
5	30	GP trainee	Female	38
6	40	PCM student	Female	45
7	35	Self-employed GP	Female	40
8	35	Employed GP	Female	40
9	35	PA student	Male	23
10	40	Self-employed GP	Male	36
11	20	MPA	Female	43
12	40	Other (clinic management)	Female	42
13	25	GP trainee	Male	32
14	60	GP trainee	Female	35
15	30	PA student	Female	29

### Core values of primary care

The interviewees emphasized that primary care should be characterized by patient-centredness:

*‘That’s really*,* I think*,* the be-all and end-all*,* that I know the patients*,* that you know the patient in all areas of life in order to be able to assess them.’ (GP trainee*,* female*,* 35 years)*.

According to the interviewees, it was relevant to devote sufficient time to the patients’ concerns. In this way, a trusting doctor-patient communication can be established. Continuity of patient support was also emphasized as essential. Therefore, short waiting times and good accessibility by telephone, as well as on-site, were seen as important criteria for primary care. According to the interviewees, future changes in primary care could particularly benefit patients with chronic illnesses, older people, and socially disadvantaged people. The study participants thought that the transformation to team-based care could contribute to patient-centred and continuous care, integrating biopsychosocial aspects and focusing more on prevention:

*“I think the trust building would be greater if you know we are here as a team and we are looking out for you. […] So now it would be more the physical-mental level*,* not purely the medical expert knowledge. I would leave that with specialists*,* and I wouldn’t include them [into a team-based concept]*,* because I think that would go beyond the scope. Exactly*,* rather better*,* really better primary care.“ (GP trainee*,* female*,* 35 years)*.

As prerequisites for good future primary care, secure funding and fair remuneration for the entire practice team were suggested by the interviewees:

*‘However*,* the remuneration must also be such that good family medicine is actually worthwhile*,* in that I can give patients half an hour of time if necessary. The fact that this half an hour is completely beyond the remuneration that we receive on a quarterly basis is a problem.’ (employed GP*,* male*,* 38 years)*.

Regular staff training and evidence-based care were also cited as quality features of primary care. The use of step-by-step diagnostics and the avoidance of unnecessary or incorrect treatments were further mentioned as relevant aspects.

The importance of cooperation with other healthcare facilities and professional groups, as well as the involvement of the entire practice team, was also emphasized. According to the interviewees, collaboration between different professional groups and medical disciplines could lead to a pooling of knowledge and improved patient management. Clear and interest-based role allocations within the team and its network were seen as beneficial. Increased networking with other healthcare providers - ideally under one roof, otherwise digitally - and targeted management of patients were mentioned as possible ways to optimize care:

*‘If it’s really all under one roof*,* I think that’s a dream of every medical professional or physician and even patients*,* if you practically have a one-stop-shop option*,* I think that’s the best there can be.’ (PA student*,* male*,* 23 years)*.

The interviewees mentioned that digital tools could be used, especially to facilitate administration and communication. Increased use of digital tools to monitor patients was seen as both a potential advantage, as it can improve interprofessional work and communication, and a critical factor, as there might be technical difficulties.

Considering the benefits as well as the challenges of the transformation to team-based care, the participants think that this concept could support a reduction in incorrect treatment and cost savings in the healthcare system in the end:

*‘I am actually firmly of the opinion that if we can provide patients with optimal care via team-based concepts*,* via these networks*,* we could save costs for the healthcare system because we could simply recognize secondary diseases in time or perhaps even prevent them.’ (PCM student*,* female*,* 45 years)*.

The core values mentioned in the interviews, as well as the methods and necessary competencies suggested for their implementation, along with possible outcomes, are summarized in Table 2.

**Table 2:** Core values and suggestions for implementation, as well as expected outcomes according to the interviewees

Mentioned core value	Suggested methods and competencies for the implementation of the core values	Expected outcomes (besides patient health)
Patient-centred care	• Enough time for patients • Trusting relationship • Holistic perspective • Comprehensive care	• Patient satisfaction • Prevention and health promotion
Continuity of care	• Accessibility of care • Short waiting time • Monitoring (via digital tools)	• Patient satisfaction • Prevention
Professionalism and Excellence	• Evidence-based practice • Regular trainings • Step-by-step diagnostics	• Avoidance of unnecessary and incorrect treatments
Interprofessional collaboration	• Clear and interest-based role allocations • Increased networking • Digital tools for administration and communication	• Pooling of knowledge • Improved patient management/coordination • Cost savings

### Concepts

Many interviewees already perceived their own practice as a team practice, at least to some extent. For some, the term ‘team’ referred to the interprofessional composition of the team, while for others, it referred to the type of work, characterized by low hierarchies and high flexibility, or a ‘team feeling’.

Out of 15 people, five had already experienced other concepts of primary care in other parts of Germany or abroad. Positive experiences were made in Switzerland, in the United Kingdom, and in an anthroposophical clinic in Germany. Ambivalent experiences were made in France.

Besides personal experiences, the interviewees had theoretical knowledge about primary care concepts in other countries and Germany. Positively rated examples were Switzerland, Sweden, New Zealand, and the Netherlands. The concept of the HÄPPI pilot program from the German federal state of Baden-Württemberg [11] was also rated positively. In the United Kingdom, both positive and negative aspects were mentioned. Respondents rated aspects of the primary care system in the USA and Italy rather negatively. The primary care concepts mentioned, along with their positively or negatively rated aspects, are presented in Table 3.

**Table 3:** Subjective rating of aspects in primary care concepts by study participants (sorted alphabetically by country)

Country	Positively rated aspects of the primary care system	Negatively rated aspects of the primary care system
Canada	• primary physician system and gatekeeping	
Denmark	• high degree of digitalisation	
France	• less complex system • digitalisation and digital patient records • GPs can concentrate on medical tasks	• GPs often work alone without employees
Germany	• interprofessional and integrative care in some clinics • HÄPPI concept • routine diagnostics by PAs	
Italy		• no functioning gatekeeping, patients often go directly to hospitals
Netherlands	• community nurses • gatekeeping • digitalisation	
New Zealand	• community nurses with a lot of responsibility	
Sweden	• gatekeeping • regular trainings • good remuneration of GPs • community nurses • high degree of digitalisation (digital patient records, telemedicine) • good network between GPs and other medical specialists • delegation is clearly regulated • good reconciliation of family and medical career	
Switzerland	• good coordination between outpatient and inpatient care • interdisciplinary case conferences • telemedicine	
United Kingdom	• involvement of non-physician professional groups (e.g. chest pain nurses) • examinations in pharmacies (blood sampling and blood pressure measurement) • gatekeeping	• long waiting times for appointments • first contact with non-physicians can be problematic with severe symptoms
USA		• focus on profit maximisation • private payments necessary • health insurance not obligatory

Overall, the respondents were in favour of GP-centred care in the sense of the primary physician principle and gatekeeping:

*‘I think that the GP-centred concept is really at the forefront. […] In my view*,* GP-centred care is simply essential and the only thing that really makes sense at the primary care level.’ (self-employed GP*,* female*,* 40 years)*.

Reasons for the preference of GP-centred care were the improved patient coordination as well as the promotion of a preventive focus when combined with a non-fee-for-service provider payment, for example in Sweden:

*‘If you compare this with the Swedish system*,* for example*,* there is a very strong primary physician system where patients are forced to go to their local general practitioner. So*,* there is primarily no free choice of physician there*,* and referrals are the only way to see a specialist. And the remuneration is structured in such a way that the GP*,* for example*,* simply gets the money for looking after these 1000 people. This means that if they are rarely ill*,* the GP has less work*,* but he or she still earns the same. If the GP doesn’t do such a good job*,* they often have to present themselves to him and he has a worse structure.’ (employed GP*,* male*,* 38 years)*.

### Measurement of best practice criteria

To evaluate the quality of future primary care concepts, respondents identified various measurable criteria. On the one hand, these included subjective criteria such as patient satisfaction and the perceived quality of treatment (Patient-Reported Experience Measures, PREMs). On the other hand, measurable clinical parameters of chronically ill patients can be examined in comparative studies between conventional and new, innovative primary care concepts, such as Patient-Reported Outcome Measures (PROMS). In addition, further criteria could be analysed from the patients’ perspective (waiting times, duration of treatment, availability of medication) as well as from the perspective of the practice staff (satisfaction of employees, days absent, turnover, number of emergencies).

## Discussion

### Summary of main results

This study included *n* = 15 participants primarily working in primary care as GP trainees, employed or self-employed GPs, MPAs, PCM or PA students, or other health professionals (mean age = 36.13 years (SD = 6.13), 66.67% female), with six months to over 20 years of experience. The interviewees emphasised patient-centred and continuous care as core values of primary care and highlighted the need for sufficient time for their patients to establish a trusting relationship. Interprofessional team-based care was perceived as beneficial, particularly for patients with chronic illnesses and those from disadvantaged backgrounds. Participants valued interprofessional collaboration, evidence-based practice, and digital tools, though technical challenges were noted. Secure funding and fair remuneration were reported as prerequisites for high-quality primary care.

The study participants favoured primary care concepts that focused on GP-centred care with gatekeeping, a high degree of digitalisation, cooperation with non-physician health professionals such as community nurses, clear role allocations, and good coordination in interprofessional and intersectoral networks. Furthermore, it was considered important by the study participants that their medical career is compatible with family life. To evaluate future GP models, they recommended PREMs, PROMs, and operational metrics such as waiting times, staff satisfaction, and financial aspects.

### Interprofessional teams to support patient-centredness and continuity of care

Patient-centredness and continuity of care emerged as the most frequently mentioned and relevant core values in primary care. An interprofessional, team-based approach may support both. Evidence suggests that team-based care can enhance patient-centredness, provided that strong intra-team relationships exist [21]. Contrary to concerns raised in the study by Arvidsson, et al. [1] our interviewees viewed interprofessional teams as potentially strengthening rather than fragmenting continuity of care. This is supported by Hustoft, et al. [22], who found that patients treated by interprofessional rehabilitation teams with higher relational coordination reported improved continuity of care and better outcomes in daily activities. Similarly, patients from Norway reported that team-based care improved the time available for care, the educational content of consultations, and continuity of care overall [23]. These findings suggest that team-based care can enhance continuity of care from the patient’s perspective, underscoring the importance of effective communication and collaboration among healthcare providers. Health professionals also emphasize that continuity depends on interdisciplinary collaboration, which in turn depends on stable organizational frameworks and robust knowledge-sharing mechanisms, as shown in a qualitative study by Ljungholm, et al. [24]. Ensuring continuity in primary care requires regular communication, clearly defined roles, mutual trust, and shared goals within interprofessional teams [25].

### Core values of primary care in Germany and Europe

The German Society for General Practice and Family Medicine (DEGAM) defined core values in 2002 that remain relevant today, including holistic, patient-centred care with a hermeneutic approach, continuity, excellence and professionalism (referring to evidence-based medicine), community orientation, and interprofessional collaboration [26]. Our findings align mostly with these values. However, community orientation was not explicitly mentioned in our interviews, as this aspect mainly was seen as part of patient-centredness. As noted by Arvidsson, et al. [1], an update of these core values may be timely. This update could, for example, include interprofessional care with clearly defined roles and the role of digitalisation in supporting team communication and administration. These aspects were perceived as important by the professionals we interviewed. Moreover, inter- and transdisciplinary collaboration is seen as essential to address complex challenges such as the climate crisis, a point already included in the revised European definition of general practice [27]. While the current DEGAM definition highlights patient protection and the avoidance of over-, under-, and misprovision, our interviewees also identified patient satisfaction and experience of care as important quality indicators. Interestingly, only one-third of GP trainees in Germany report having studied core values in the past [28]. To support reflective practice and high-quality primary care, these values should be introduced early in general practice education [28].

### Best practices

Examining interprofessional collaboration models already implemented in other European countries could provide valuable insights for identifying best practices of such care concepts in Germany. Our interviewees frequently referred to models characterized by a high degree of interprofessional collaboration, advanced digitalization, and gatekeeping as best practices for primary care. Such approaches can be found in countries like Sweden, Switzerland, and the Netherlands. For example, Dutch primary care models for managing multimorbidity emphasize structured care protocols, regular interprofessional meetings, and comprehensive quality management [29]. These elements have been shown to improve care quality and efficiency and are currently being tested in a pilot project in the German county of Baden-Württemberg [11].

### Prerequisites of value-based primary care

While core values can be seen as intrinsic factors for delivering value-based primary care within a team-based concept of care, remuneration can be viewed as an extrinsic factor [30]. The participants of our study highlighted the need for secure funding and fair remuneration as prerequisites for value-based primary care. Increasing financial support of primary health care in general is a policy recommendation by De Foo, et al. [10] to achieve the goal of comprehensive care.

A rapid review by Aggarwal, et al. [30] compared different payment models and emphasized the importance of transitioning away from fee-for-service schemes. Salary-based models, where professionals receive fixed compensation regardless of the volume of services provided, foster teamwork by eliminating individual financial incentives. Quality-based payment models, which reward outcomes such as patient satisfaction or improved health, further support collaboration. In contrast, fee-for-service models may undermine teamwork by encouraging competition and individual performance over shared goals.

### Evaluation of concepts of care

To evaluate new primary care models, interviewees highlighted several measurable aspects, primarily patient-reported measures. Including patient perspectives is essential to ensure that care remains patient-centred and addresses individual needs.

Patient health status as well as patient experience are seen as indicators to measure the performance of interprofessional team-based care [31]. The study of Sturm, et al. [32] highlights that patients value effective communication, continuity, and coordination in primary care. Positive experiences are linked to personal and functional information flow, reassurance from non-physician staff, and the development of long-lasting doctor-patient relationships. Interprofessional teams support these aspects by facilitating comprehensive care, improving information flow, and providing consistent support, which enhances overall patient satisfaction and outcomes.

Instruments that assess communication quality, respectful interaction, and shared goals can help evaluate team performance. Shoemaker, et al. [33] identified 48 such tools suitable to assess team performance in primary care but emphasized the need for further adaptation and validation in this context.

### Strengths and limitations

A key strength of this study is the inclusion of diverse health professionals, ensuring a broad range of perspectives. The qualitative and open approach enabled an exploration of the values of primary care and best practice criteria. To minimize interviewer bias, two of four members of the research team conducted each interview, alternating between moderation and taking minutes.

However, the study has some limitations. This study is based on 15 individual interviews. While recurring views and overlapping content emerged during data collection, suggesting a degree of saturation, the small sample size should be considered when interpreting the findings. As the analysis relied solely on deductive coding based on prior research, context-specific categories may have been overlooked, which could have been captured through complementary inductive coding. The patient perspective was not included, but should be addressed in pilot projects on team-based care in Germany, as it was done, for example, in Norway [23] and suggested by our interview participants. Additionally, the sample consisted mostly of younger participants, whose perspectives are relevant for the future of primary care and the establishment of team-based practices. Thus, conclusions regarding the views of older health professionals cannot be drawn. Furthermore, as the study focused on Bavaria, the findings may not be fully generalisable to other regions.

## Conclusions

Our findings suggest that core values of primary care—particularly patient-centredness and continuity—can be strengthened through interprofessional teamwork. While these values act as intrinsic motivators for high-quality care and clinical decisions, extrinsic factors such as fair remuneration and incentives for collaboration are equally important. Best practices for interprofessional teamwork in primary care include clear role allocations, fair remuneration, and a high degree of digitalisation to facilitate administration and communication. For the evaluation of care concepts, the patient perspective is particularly relevant, considering both patient health and patient experience. In addition to the patient perspective, the satisfaction of medical staff and team performance are further important indicators.

## Supplementary Information

Supplementary Material 1.

Supplementary Material 2.

Supplementary Material 3.

## Data Availability

The datasets generated and analysed during the current study are not publicly available due to ethical considerations and data protection regulations.

## Abbreviations

GPs: General practitioner/
GP: General practice
MAs: Medical assistants
PROM: Patient–reported outcome measure
PREM: Patient–reported experience measure
VERAH: Versorgungsassistenz in der Hausarztpraxis (care assistant in general practice)
NäPa: Nichtärztliche Praxisassistenz (non–physician practice assistant)
PA: Physician assistant
PCM: Primary care management
HÄPPI: Hausärztliches Primärversorgungszentrum– Patientenversorgung interprofessionell (general practice primary care centers – interprofessional patient care)
