# Supplementary material for: Core values and best practice criteria for interprofessional teams in primary care: a qualitative interview study with general practitioners and other health professionals from Bavaria, Germany
Source: BMC Prim Care. 2025 Dec 11;27:28. doi: 10.1186/s12875-025-03114-3 (PMC12853866; doi:10.1186/s12875-025-03114-3)
Supplement: Supplementary file 2 — Supplementary Material 2. [file 12875_2025_3114_MOESM2_ESM.docx]

Supplement 2: COREQ Checklist (Consolidated Criteria for Reporting Qualitative Research)

| Domain | Item | Guide Questions/Description | Statement |
| --- | --- | --- | --- |
| Domain 1: Research team and reflexivity | Interviewer/facilitator | Which author/s conducted the interview or focus group? | KZ, DW, MT, LS |
|  | Credentials | What were the researcher's credentials? e.g. PhD, MD | Health scientists (KZ: MPH, MT: MSc) and physicians (DW, LS) |
|  | Occupation | What was their occupation at the time of the study? | Scientists |
|  | Gender | Was the researcher male or female? | Female (KZ, MT, LS) and male (DW) |
|  | Experience and training | What experience or training did the researcher have? | KZ and MT have expertise in conducting qualitative interview studies, LS and DW performed a qualitative interview study for the first time under instructions of MT and KZ |
|  | Relationship established | Was a relationship established prior to study commencement? | There was E-Mail contact with the study participants and a focus group study took place before conducting the individual interviews. |
|  | Participant knowledge of the interviewer | What did the participants know about the researcher? e.g. personal goals, reasons for doing the research | The study participants got a study information including study aims and gave written informed consent to participate in the study |
|  | Interviewer characteristics | What characteristics were reported about the interviewer/facilitator? e.g. bias, assumptions, reasons and interests in the research topic | It was reported that the interviewer was a scientist of the institute of general practice at the university of Augsburg. |
| Domain 2: Study design | Methodological orientation and theory | What methodological orientation was stated to underpin the study? e.g. grounded theory, discourse analysis, ethnography, phenomenology, content analysis | Content analysis (framework method) |
|  | Participant selection | How were participants selected? e.g. purposive, convenience, consecutive, snowball | Participants who had demonstrated high engagement or relevant experience during the focus groups—such as interest in innovation or familiarity with alternative care models—were preferentially selected for individual interviews. At least one person from each professional group (employed GPs, self-employed GPs, GP trainees, MAs/care assistants, PA students, PCM students, and other health professionals) was contacted. |
|  | Method of approach | How were participants approached? e.g. face-to-face, telephone, mail, email | Video-conference tool Zoom |
|  | Sample size | How many participants were in the study? | n = 15 |
|  | Non-participation | How many people refused to participate or dropped out? Reasons? | Of all individuals contacted for the individual interviews, n = 6 did not participate due to loss of interest or scheduling difficulties. |
|  | Setting of data collection | Where was the data collected? e.g. home, clinic, workplace | Audio data were recorded using an offline device. Both audio and transcribed files were stored on a secure server at the authors’ institution. Audio files were deleted after completion of the analysis. |
|  | Presence of non-participants | Was anyone else present besides the participants and researchers? | No |
|  | Description of sample | What are the important characteristics of the sample? e.g. demographic data, date | See table 1 |
|  | Interview guide | Were questions, prompts, guides provided by the authors? Was it pilot tested? | The interviews were semi-structured and the researchers used an interview guide that was pretested. |
|  | Repeat interviews | Were repeat interviews carried out? If yes, how many? | No |
|  | Audio/visual recording | Did the researchers use audio or visual recording to collect the data? | The researchers used audio recordings. |
|  | Field notes | Were field notes made during and/or after the interview or focus group? | The researcher responsible for taking minutes also recorded field notes when necessary, such as documenting technical issues or the atmosphere during the interviews. |
|  | Duration | What was the duration of the interviews or focus group? | See table 1 |
|  | Data saturation | Was data saturation discussed? | Preliminary analyses were conducted while interviews were still ongoing, during which recurring views and overlapping themes began to emerge, indicating a degree of thematic saturation. |
|  | Transcripts returned | Were transcripts returned to participants for comment and/or correction? | No |
| Domain 3: Analysis and findings | Number of data coders | How many data coders coded the data? | Each interview was coded and double-checked by two research team members |
|  | Description of the coding tree | Did authors provide a description of the coding tree? | See Additional file 1 |
|  | Derivation of themes | Were themes identified in advance or derived from the data? | Themes were identified in advance (deductive approach). |
|  | Software | What software, if applicable, was used to manage the data? | Max QDA and Excel |
|  | Participant checking | Did participants provide feedback on the findings? | No |
|  | Quotations presented | Were participant quotations presented to illustrate the themes/findings? Was each quotation identified? | Yes (see results section) |
|  | Data and findings consistent | Was there consistency between the data presented and the findings? | Yes (see results section) |
|  | Clarity of major themes | Were major themes clearly presented in the findings? | Yes (see results section) |
|  | Clarity of minor themes | Is there a description of diverse cases or discussion of minor themes? | Yes (see results section) |

Tong A, Sainsbury P, Craig J: Consolidated criteria for reporting qualitative research (COREQ): a 32-item checklist for interviews and focus groups. International journal for quality in health care 2007, 19(6):349-357.
