# Supplementary material for: Core values and best practice criteria for interprofessional teams in primary care: a qualitative interview study with general practitioners and other health professionals from Bavaria, Germany
Source: BMC Prim Care. 2025 Dec 11;27:28. doi: 10.1186/s12875-025-03114-3 (PMC12853866; doi:10.1186/s12875-025-03114-3)
Supplement: Supplementary file 3 — Supplementary Material 3. [file 12875_2025_3114_MOESM3_ESM.docx]

**Additional file 3: Analysed sections of the interview questionnaire of the individual interviews**

*Introductory words, project description, declaration of rights*

**Demographic information**

At the beginning we would like to ask you to briefly introduce yourself: age, professional experience, everyday life.

*Questions regarding the focus group interviews conducted prior to the individual interviews, as well as personal views on the working environment, communication, organisation, and personnel management (not relevant to this study).*

**Concepts of primary care**

Are you currently working in an interprofessional practice? Would you consider your practice to be a team-based model?

If so, could you briefly describe the concept of care?

If yes, what positive or negative experiences have you had?

Have you encountered other concepts of primary care abroad or in other regions of Germany?

If yes, which ones? Could you briefly describe them?

If yes, how did you come to know about these models (e.g. through your own work experience, as a patient, etc.)?

If yes, what were your positive or negative experiences?

Do you have any family members, friends, or acquaintances who have experienced different care models in other countries or in other regions of Germany?

If yes, which ones? Could you briefly describe them?

If yes, how did they become familiar with the models (e.g. through work, as a patient, etc.)?

If yes, what were their positive or negative experiences?

Are you aware of any theoretical concepts in which various healthcare sectors are integrated?

If so, which ones, and where did you come across them (e.g. involved in development, read about them, heard about them, etc.)?

**Best Practice Criteria**

What qualities or features should a future general practice have, in your opinion?

What do you understand by good general practice/ family medicine?

Optionally: In what ways could restructuring into a team-based practice contribute to good or improved primary care?

**Closing**

Are there any further comments or points you would like to add on this topic?

*Acknowledgements and closing remarks*
